# The Metalloprotease Meprinβ Processes E-Cadherin and Weakens Intercellular Adhesion

**DOI:** 10.1371/journal.pone.0002153

**Published:** 2008-05-14

**Authors:** Maya Huguenin, Eliane J. Müller, Sandra Trachsel-Rösmann, Beatrice Oneda, Daniel Ambort, Erwin E. Sterchi, Daniel Lottaz

**Affiliations:** 1 Institute of Biochemistry and Molecular Medicine, University of Bern, Bern, Switzerland; 2 Molecular Dermatology, Institute of Animal Pathology, University of Bern, Bern, Switzerland; University of Arkansas, United States of America

## Abstract

**Background:**

Meprin (EC 3.4.24.18), an astacin-like metalloprotease, is expressed in the epithelium of the intestine and kidney tubules and has been related to cancer, but the mechanistic links are unknown.

**Methodology/Principal Findings:**

We used MDCK and Caco-2 cells stably transfected with meprinα and or meprinβ to establish models of renal and intestinal epithelial cells expressing this protease at physiological levels. In both models E-cadherin was cleaved, producing a cell-associated 97-kDa E-cadherin fragment, which was enhanced upon activation of the meprin zymogen and reduced in the presence of a meprin inhibitor. The cleavage site was localized in the extracellular domain adjacent to the plasma membrane. *In vitro* assays with purified components showed that the 97-kDa fragment was specifically generated by meprinβ, but not by ADAM-10 or MMP-7. Concomitantly with E-cadherin cleavage and degradation of the E-cadherin cytoplasmic tail, the plaque proteins β-catenin and plakoglobin were processed by an intracellular protease, whereas α-catenin, which does not bind directly to E-cadherin, remained intact. Using confocal microscopy, we observed a partial colocalization of meprinβ and E-cadherin at lateral membranes of incompletely polarized cells at preconfluent or early confluent stages. Meprinβ-expressing cells displayed a reduced strength of cell-cell contacts and a significantly lower tendency to form multicellular aggregates.

**Conclusions/Significance:**

By identifying E-cadherin as a substrate for meprinβ in a cellular context, this study reveals a novel biological role of this protease in epithelial cells. Our results suggest a crucial role for meprinβ in the control of adhesiveness via cleavage of E-cadherin with potential implications in a wide range of biological processes including epithelial barrier function and cancer progression.

## Introduction

Meprin is a zinc metalloendopeptidase of the astacin family, which was first discovered as an abundantly expressed protease at the brush border membrane of epithelial cells in the kidney [1] and the intestine [2]. Meprin consists of two homologous subunits, meprinα and meprinβ, which share 42% amino acid sequence identity. The α and β subunits form homo- or heterodimers covalently linked via disulfide bonds, as well as higher multimeric structures resulting from non-covalent association of dimers [3]. Both subunits are synthesized as zymogens and targeted to the cell surface. The multimers composed of the β subunits are mainly transmembrane complexes, whereas those composed only of meprinα are secreted, due to an intracellular processing event that removes the transmembrane anchor [4]–[6]. Their activation requires removal of a propeptide by enzymes such as trypsin or plasmin [7], [8]. These proteases cleave a wide range of substrates *in vitro*, including bioactive peptides (angiotensins, TGF-α), growth factors and hormones (gastrin, glucagon) [2], [9], cytokines (monocyte chemoattractant protein-1, IL-1β) [10] and extracellular matrix proteins (laminin 1 and 5, type IV collagen, fibronectin) [11], [12]. However, meprinα and meprinβ display different substrate repertoires and cleavage site specificities [13].

The broad proteolytic activity of meprin indicates multiple functions in health and disease. Indeed, meprin has been linked to both cancer and inflammation [14]–[16]. Expression of meprinα and meprinβ has been demonstrated in different types of cancer cells, including colorectal, breast, osteosarcoma and pancreatic cell lines [17], [18]. Meprinα is aberrantly secreted to the stroma in colorectal cancer [14]. However, the substrates and pathogenetic role of meprins in cancer are not known. Here we describe the tumor suppressor E-cadherin as a novel target of meprinβ.

E-cadherin is a transmembrane glycoprotein and the principal component of adherens junctions in epithelial cells. It mediates homophilic calcium-dependent interactions between adjacent cells, which are stabilized by intracellular association with the catenin proteins β-catenin, plakoglobin, p120-catenin and α-catenin. These associations contribute to the regulation of diverse cellular processes such as cell polarization, aggregation and migration [19]. In cancer, downregulation of E-cadherin has been shown to play a crucial role in the progression of well differentiated adenoma to invasive carcinoma [20], and E-cadherin is therefore considered to act as a tumor suppressor [21].

Loss or alteration of E-cadherin has been demonstrated in adenocarcinomas of many tissues including kidney and intestine [22]–[28], and cleavage of E-cadherin has been described as one of the cellular mechanisms controling its function. For example, the metalloproteases stromelysin-1 (MMP-3), matrilysin (MMP-7) or ADAM-10 cleave E-cadherin extracellularly, generating a soluble fragment that impairs cell-cell adhesion [29], [30]. We demonstrate that meprinβ cleaves the ectodomain of E-cadherin, which correlates with a reduction in intercellular adhesive strength and cell aggregation in meprinβ-expressing cells.

## Methods

### Antibodies and recombinant proteins

Meprinβ-specific and meprinα/β-crossreactive rabbit polyclonal antibodies were generated using a glutathion S-transferase fusion protein expressed in E. coli, as previously described [4], [31]. Antibodies recognize meprin zymogens and activated forms. Anti-E-cadherin antibodies used in this study were: DECMA (Sigma, St. Louis, MO, U.S.A.), SC7870 (Santa Cruz Biotechnology, Heidelberg, Germany) and C-terminal clone 36 (BD Transduction Laboratories, San Jose, U.S.A.). Anti-β-catenin (clone 14) and anti-plakoglobin (clone 15) monoclonal antibodies were from BD Transduction Laboratories. Monoclonal anti-tubulin antibody (Clone TUB-1A2) was from Sigma. Horseradish peroxidase-conjugated anti-rabbit, anti-mouse and anti-rat secondary antibodies were obtained from Amersham Biosciences (Buckinghamshire, U.K.) and Dako Cytomation (Denmark). Active recombinant meprinα and meprinβ were purified from a baculovirus expression system in insect cells as described previously [32]. Purified recombinant human ADAM-10 (active ectodomain derived from an insect cell expression system) and pro-MMP-7 (expressed in a mouse myeloma cell line) were from R+D Systems, Abingdon, U. K. Pro-MMP-7 was activated with 1 mM p-amino-phenylmercuric acetate (AMPA) at 37°C for 1 h.

### Cell culture

Culture media and supplements were obtained from Invitrogen (Basel, Switzerland). Actinonin, bovine trypsin and soybean trypsin inhibitor were purchased from Sigma. Wild-type MDCK, MDCKα, MDCKβ and MDCKαβ cells [5]–[7] were grown in minimal essential medium (MEM) supplemented with 25 mM HEPES, 5% fetal bovine serum and 2 mM glutamine. Wild-type Caco-2 cell subclone “TC7”, a generous gift from Prof. Monique Rousset, and the meprinβ-expressing clone “β21” were grown in Dulbecco's Modified Eagle Medium (DMEM) supplemented with 20% fetal bovine serum, 2 mM glutamine, 4.5 g/l D-glucose and non-essential amino acids (100 µM each). Cells were seeded at a density of 8×10^5^ on polyethylene terephtalate (PET) cell culture inserts (4.2 cm^2^ growth area, 0.4 µm pore size, from BD Biosciences, Basel, Switzerland) and incubated in 2 ml apical medium and 3 ml basolateral medium. Establishment of cell monolayers was followed by phase contrast microscopy. Prior to harvesting, subconfluent and confluent cell cultures were incubated for 24 hours in serum-free medium (Advanced minimum essential medium, AdvMEM). For meprin *in situ* activation, cell cultures were treated with bovine trypsin (10 µg/ml in AdvMEM) for 30 min, washed twice with phosphate-buffered saline and subsequently incubated with fresh AdvMEM containing soybean trypsin inhibitor (20 µg/ml). For *in situ* meprin inhibition, cells were incubated with 100 nM actinonin in AdvMEM for 24 hours.

Stable meprinβ-expressing Caco-2 cells were generated using METAfectene transfection reagent (Invitrogen, Basel, Switzerland) according to the manufacturer's protocol. Caco-2-TC7 cells were grown in 6 cm dishes until 50% confluence and subsequently incubated with 2 ml OPTI-MEM containing the transfection cocktail with 10 µg meprinβ-expression plasmid [7] and 2 µg pBKCMVneo^r^ (Stratagene, La Jolla, CA, U.S.A.) for 6 h. Cell cultures were supplemented with additional 2 ml complete medium for an overnight incubation, passaged from each 6 cm dish into five 10 cm dishes and selected in complete medium with 1000 µg/ml and 700 µg/ml geneticin for the two following weeks. Caco-2 cell clones were harvested using cloning cylinders and screened for meprinβ expression using Western blot analysis.

### Preparation of cell lysates, SDS-PAGE and immunoblotting

Cells were washed twice with phosphate-buffered saline and lysed on ice in 25 mM Tris-HCl pH 8, 50 mM sodium chloride, 1% IGEPAL (Sigma), 1% sodium deoxycholate (Sigma), with complete protease inhibitor cocktail EDTA-free (Roche, Basel, Switzerland) for 45 minutes. Cell debris was removed by centrifugation and the protein content in the resulting extracts was determined using the bicinchoninic acid (BCA) protein assay (Pierce, Rockford, U.S.A.). All chemicals for gel electrophoresis were obtained from Bio-Rad Laboratories AG (Reinach, Switzerland). Equal protein amounts (15 µg) were solubilized by boiling in Laemmli buffer, subjected to electrophoresis under reducing conditions on 10% SDS-polyacrylamide gels and subsequently electroblotted onto a polyvinylidene difluoride (PVDF) membrane (Hybond P, Amersham Biosciences). Subsequent incubations were done using 25 mM Tris-HCl pH 7, 150 mM sodium chloride, 2.5 mM potassium chloride, 0.1% Tween-20 (TBS-T) at room temperature. After saturation with TBS-T containing 5% dry milk, the membrane was incubated with the first antibody overnight and the appropriate horseradish peroxidase-conjugated secondary antibody for 2 hours. Immune complexes were visualized on X-ray films using enhanced chemiluminescence (ECL Plus, from Amersham Biosciences). The membrane was stripped in 62.5 mM Tris-HCl pH 6.8, 2% sodium dodecyl sulfate (SDS), 50 mM dithiothreitol (DTT) on a shaking plate at 65°C for 45 minutes and washed extensively in TBS-T. The efficiency of stripping was ascertained by exposing a X-ray film on the membrane prior to reprobing for another target protein. Apparent molecular weights were determined using a calibration plot with standard proteins as a reference (broad range SDS-PAGE Standards, Bio-Rad Laboratories).

### Immunoprecipitation

The anti-E-cadherin antibody (DECMA, 6 µg) was preabsorbed to 60 µl protein G-Sepharose beads (50% slurry, from Amersham Biosciences) in 500 µl cytoskeleton (CSK)-buffer (10 mM piperazine-N,N′-bis(2-ethane-sulfonic acid) pH 6.8, 300 mM sucrose, 50 mM sodium chloride, 3 mM magnesium chloride, 0.5% (v/v) Triton X-100 (Sigma), 1.2 mM phenylmethylsulfonyl fluoride (Sigma), 10 mM sodium pyrophosphate, 20 mM sodium fluoride (Sigma), with complete protease inhibitor cocktail EDTA-free (Roche)) at 4°C for 2 hours. The beads were washed twice with 500 µl CSK-buffer, resuspended in CSK-buffer to create a 50% slurry and combined with precleared 500 µg cellular protein extract (in 500 µl CSK-buffer) or 1.2 ml cell culture supernatant. E-cadherin was immunoprecipitated at 4°C for 4 hours. Supernatants after immunoprecipitation were analyzed as controls. Beads were washed once with meprin-assay buffer (50 mM Tris-HCl pH 7.5, 1 mM magnesium chloride with complete protease inhibitor cocktail EDTA-free (Roche), and either analyzed by SDS-PAGE and Western blotting, or resuspended in 100 µl meprin assay buffer and divided into aliquots for *in vitro* cleavage reactions (see below).

### E-cadherin *in vitro* cleavage assay with human recombinant meprin, ADAM-10 and MMP-7

Protein extracts from MDCK cells (50 µg) or immunoprecipitated E-cadherin were incubated with meprinα, meprinβ, ADAM-10 or MMP-7 at 37°C in corresponding optimized assay buffers supplemented with complete protease inhibitor cocktail EDTA-free (Roche). The assay buffer for cleavage reactions with meprinα and meprinβ was 50 mM Tris-HCl pH 7.5, 1 mM magnesium chloride (Meprin assay buffer) supplemented with 1 mM calcium chloride. Assay buffers for ADAM-10 and MMP-7 were 25 mM Tris-HCl pH 9.0, 2.5 µM zinc chloride, 0.005% Brij35 and 50 mM Tris-HCl pH 7.5, 10 mM calcium chloride, 150 mM sodium chloride, 0.05% Brij35, respectively. Cleavage reactions were stopped by adding 10 mM EDTA. Beads were washed once in corresponding assay buffer. E-cadherin cleavage fragments were separated on 10% SDS-polyacrylamide gels and detected by Western blotting.

### Immunofluorescence staining and confocal laser scanning microscopy

MDCK cells grown on filter inserts were washed twice with phosphate-buffered saline, fixed in 2.5% paraformaldehyde in phosphate buffer saline for 30 minutes and permeabilized with 0.1% Triton-X-100 in 25 mM Tris-HCl pH 7.5, 140 mM sodium chloride (TBS) for 5 minutes To retrieve meprinβ epitopes, samples were microwaved in 10 mM citrate-monohydrate, pH 5.65 for 5 minutes. Subsequent incubations were done in TBS at room temperature. Slides were blocked in 20% goat or rabbit normal serum, incubated with primary antibodies directed against meprinβ (1∶500) or E-cadherin (DECMA 1∶3600) and appropriate secondary antibodies (AlexaFluor 488-conjugated anti-rabbit 1∶1000 and AlexaFluor 594-conjugated anti-rat 1∶400, from Molecular Probes, Invitrogen). Mounted samples (Vectashield mounting medium, from Vector Laboratories Inc., Burlingame, U.K.) were analyzed with Zeiss LSM 510 Meta coupled to the confocal inverted Zeiss microscope (Axiovert 200 M; lasers: HeNe 633 nm, HeNe 543 nm, and Ar 488 nm, Zeiss, Feldbach, Switzerland).

### Calcium-switch assay

Confluent cell monolayers were subjected to the calcium switch assay [33] as follows: The cells were seeded onto filter inserts (8×10^5^ cells/4.2 cm^2^/0.4-µm pore size) and allowed to attach in Ca^2+^-containing DMEM (1.8 mM Ca^2+^) until confluence. For the switch, the cells were rinsed three times with phosphate-buffered saline and incubated in S-MEM (low Ca^2+^ minimum essential medium for suspension culture) (Invitrogen) which contains less than 5 µM total Ca^2+^, at 37°C for 30 or 120 minutes. Immediately after these time points, the cells were rinsed twice with phosphate-buffered saline, fixed in 2.5% paraformaldehyde and processed for immunofluorescence and confocal microscopy as described above.

### Dispase-based dissociation assay

The assay for measuring cell-cell adhesive strength [34] was modified as follows: MDCKwt and MDCKαβ cells were washed twice in phosphate-buffered saline and incubated in 0.6 ml of dispase (2.4 U/ml; Roche) for 45 minutes. Released monolayers were transferred to 15-ml conical tubes, washed once and resuspended in 0.6 ml phosphate-buffered saline. To apply a mechanical stress, the monolayers were subjected to 20 pipettings with an automatic pipette. After 1 minute, released single cells were counted in a 10 µl- aliquot. One well of each triplicate was treated with trypsin instead of dispase, to determine the total cell number, which was divided by the mechanically released single cells. Statistical analysis was performed on three separate experiments and statistical significance (*t*-test) was defined as P<0.05.

### Cell aggregation assay

The assay was adapted from a previous report [35]: Cells were resuspended at 5×10^5^ cells/ml in AdvMEM with or without 100 nM actinonin. Six 30-µl drops of the cell suspension were cultured as “hanging drops” on the inner surface of the lid of a 12-well culture dish. To prevent evaporation, 0.5 ml phosphate-buffered saline was placed in the bottom of each well. After an overnight incubation at 37°C, cell aggregates that had formed at the drop meniscus were photographed with a Nikon DIAPHOT 300 inverted microscope.

## Results

### Meprinβ generates a 97-kDa E-cadherin fragment in epithelial cells

**Figure 1:**
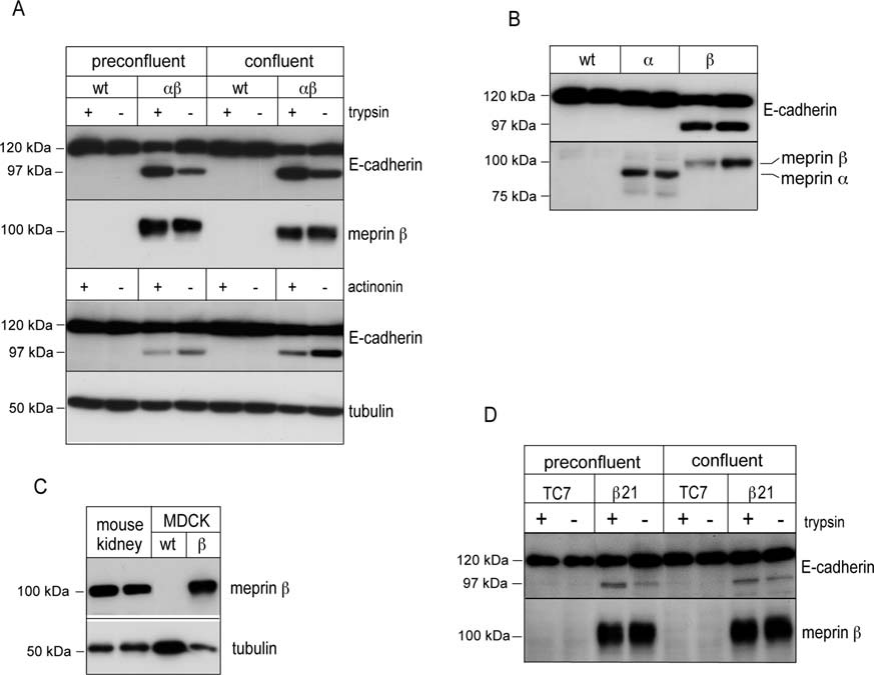
E-cadherin is truncated in MDCK and Caco-2 cells expressing meprinβ. (A,B) Wild-type and meprin-expressing cells were grown on transwell filter inserts, *in situ* treated with trypsin or actinonin (meprin activator and inhibitor, respectively), or left untreated. Immunoblot analysis of indicated cell lysates was done with a monoclonal antibody directed against the EC1 domain at the N-terminus of E-cadherin (DECMA). An additional 97-kDa fragment of E-cadherin was detected in meprinβ-expressing cells. Blots were stripped and reprobed for meprin and tubulin. (C) Protein levels of meprinβ were similar in meprinβ-expressing MDCK cells and homogenates of whole mouse kidneys (C57Bl/6 strain). 15 µg of total protein extracts were analyzed on immunoblots. (D) E-cadherin is processed in meprinβ-expressing Caco-2 cells (β21) but not in parental TC7 Caco-2 cells.

To investigate the fate of E-cadherin in the presence of meprin, we used MDCK cells as a well-established and thoroughly characterized model of renal tubular epithelial cells. MDCK cells stably expressing meprinα (MDCKα) [7], meprinβ (MDCKβ), or both meprinα and meprinβ (MDCKαβ) [5] were compared with parental wild-type cells (MDCKwt). The use of meprin-transfected epithelial cells was motivated by the lack of available cell lines expressing endogenous meprin at significant levels. Cell lysates were prepared from cells harvested at preconfluent and confluent growth stages and analyzed on Western blots. E-cadherin was cleaved in MDCKαβ cells, generating an E-cadherin fragment of 97 kDa (Figure 1A). Activation of meprin by *in situ* trypsin treatment of cell cultures increased the cleavage of E-cadherin and resulted in the accumulation of the 97-kDa fragment at the expense of the 120-kDa mature form (Figure 1A). The trypsin treatment did not affect E-cadherin in control MDCKwt cells. Conversely, inhibition of meprin activity by actinonin decreased the amount of the 97-kDa E-cadherin fragment (Figure 1A). Western blot analysis of MDCK cells expressing individual subunits revealed that meprinβ but not meprinα mediated the cleavage of E-cadherin (Figure 1B). Meprinβ protein levels in transfected MDCK cells were similar to endogenous levels in mouse kidney homogenates, excluding an artifact due to inappropriately high expression of the protease (Figure 1C).

We confirmed these observations in Caco-2 cells, a human colon carcinoma cell line which differentiates into a polarized enterocyte-like cell type and endogenously expresses meprinα [14]. E-cadherin expression was analyzed by Western blotting in the parental subclone Caco-2-TC7 in comparison to a cell clone stably expressing meprinβ (Caco-2-β21). As in MDCKαβ cells, the 97-kDa fragment was generated in meprinβ-expressing Caco-2 cells, which was enhanced after *in situ* activation by trypsin (Figure 1D). Similar levels of the 97-kDa E-cadherin fragment were detected in preconfluent and confluent cell cultures (Figure 1A, D). Taken together the findings implicate meprinβ in the proteolytic processing of endogenous E-cadherin.

### Meprinβ cleaves E-cadherin directly *in vitro*

**Figure 2:**
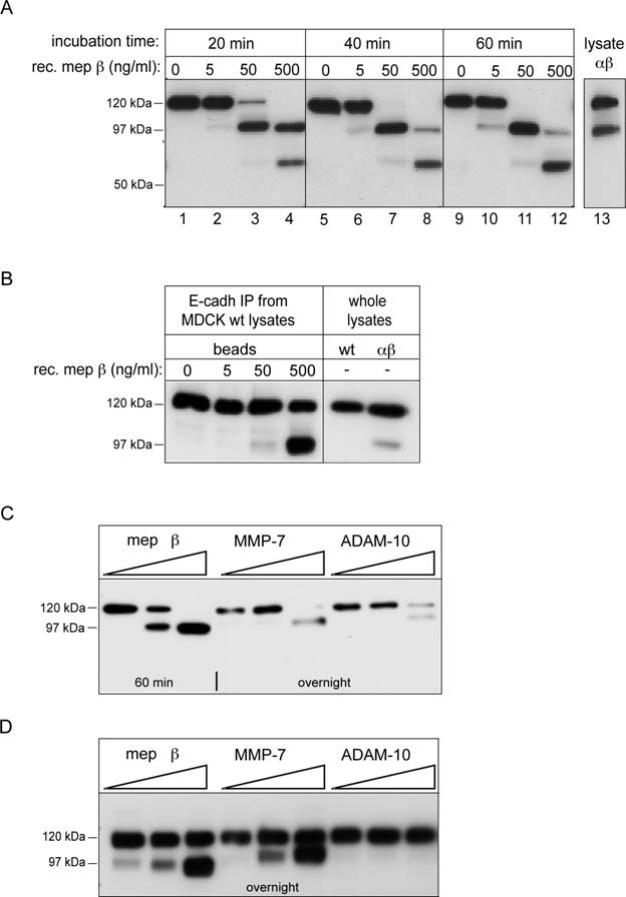
*In vitro* cleavage of E-cadherin by purified recombinant active meprinβ. (A) MDCKwt cell lysates were incubated with different concentrations of meprinβ for the indicated times. Cleavage products were analyzed on Western blots using an anti-E-cadherin antibody (DECMA). Processed E-cadherin in MDCKαβ cells are shown in comparison. (B) E-cadherin was first immunoprecipitated from MDCKwt cell lysates and subsequently incubated on the beads with recombinant meprinβ at the indicated concentration for 40 minutes. E-cadherin cleavage fragments in eluates from the beads were analyzed on immunoblots using the monoclonal N-terminal antibody (DECMA). Samples of MDCKwt and MDCKαβ cell lysates were loaded as reference. (C,D) Specific generation of the 97-kDa E-cadherin fragment by meprinβ, but not MMP-7 and ADAM-10. MDCKwt cell lysates (C) or immunoprecipitated E-cadherin on beads (D) were incubated for 1 hour or overnight with increasing concentrations of recombinant active meprinβ, MMP-7 and ADAM-10 (0.0125 nM, 0.125 nM and 1.25 nM).

To corroborate the direct cleavage of E-cadherin by meprinβ, we incubated MDCKwt cell lysates with different concentrations of the purified active recombinant protease. To avoid interference of cellular proteases, cell lysates were supplemented with protease inhibitors targeting all protease families except metalloproteases. The *in vitro* reaction was stopped at different time points and E-cadherin was analyzed by Western blotting. In the absence of meprinβ, only the mature form of E-cadherin was detectable (Figure 2A, lanes 1, 5, 9). At 5 ng/ml meprinβ, a fragment was generated that corresponded in size to the 97-kDa fragment previously observed in MDCKαβ and MDCKβ lysates (Figure 2A, lanes 2, 6, 10). At 50 ng/ml meprinβ, the 97-kDa fragment accumulated at the expense of the mature form (Figure 2A, lanes 3, 7, 11). At the highest concentration of meprinβ, the full length E-cadherin was no more detectable, and in addition to the 97-kDa fragment, a fragment of 60 kDa was also generated (Figure 2A, lanes 4, 8, 12). The assay gave identical results in the presence of the broad spectrum caspase inhibitor z-VAD-FMK (not shown), excluding any implication of these proteases known to cleave E-cadherin during apoptosis [36].

To exclude the possibility that a protease in the cell lysate processed E-cadherin downstream of meprinβ, we repeated the *in vitro* cleavage assay with immunopurified E-cadherin. Figure 2B shows that the 97-kDa E-cadherin fragment was generated upon addition of 50 ng/ml and 500 ng/ml purified meprinβ to the beads with immunoprecipitated E-cadherin.

E-cadherin has been previously reported to be cleaved extracellularly by MMP-7 and ADAM-10 [29], [30], [37]. We therefore compared the cleavage of E-cadherin with active recombinant forms of these two proteases at different concentrations *in vitro* both in MDCK cell lysate (Figure 2C) and after immunoprecipitation on beads (Figure 2D). The generated E-cadherin fragment was slightly larger than the one generated by meprinβ, indicating that MMP-7 and ADAM-10 at the enzyme concentrations used in our assay target a cleavage site C-terminal to the meprinβ site. These data provide evidence that the 97-kDa E-cadherin fragment is a different and specific product of the processing by meprinβ.

### Meprinβ colocalizes with E-cadherin in MDCK cells

**Figure 3:**
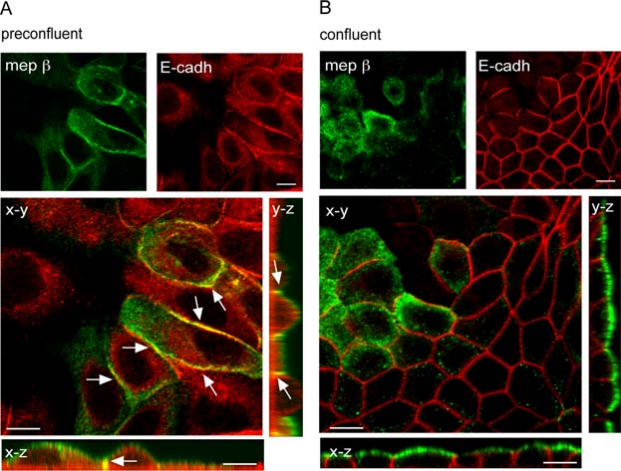
Meprinβ is partially colocalized with E-cadherin in MDCKαβ cells. The distribution of meprinβ and E-cadherin was analyzed by double immunostaining and CLSM in preconfluent (A) and confluent (B) monolayers. Pictures shown are single optical sections in the x–y axis. Corresponding orthogonal sections in the x–z and y–z axis are shown beside. Arrows in the merged picture indicate were meprinβ and E-cadherin colocalize (evidenced by the yellow color). Bar = 10 µm.

In polarized cells, meprinβ and E-cadherin are normally targeted to apical [5] and lateral membrane domains, respectively. However, our data suggest that both proteins are in contact at a certain time point. We therefore analyzed the distribution of E-cadherin and meprinβ in MDCKαβ cells in subconfluent and confluent cultures by immunofluorescence and confocal laser scanning microscopy (CLSM). In cells grown to preconfluent density, only a fraction of E-cadherin was detected at the intercellular junctions, with a concomitant pool of E-cadherin being present intracellularly (Figure 3A). Meprinβ was detected at the apical domain and in addition at lateral borders (Figure 3A). The lateral immunostaining for meprinβ was partially colocalized with the signal for E-cadherin as evidenced by the yellow color in the merged picture and the orthogonal projections shown in the x–z and y–z planes (Figure 3A). The partial colocalization of meprinβ and E-cadherin at lateral membranes indicates that E-cadherin is accessible to the protease. After formation of a confluent monolayer, the two proteins appeared to be restricted to apical and lateral domains, respectively, without microscopically distinguishable overlap (Figure 3B). However, the biochemical data shown above indicate that E-cadherin and meprinβ may still interact transiently for the processing to occur.

### Molecular mapping of the E-cadherin cleavage site

**Figure 4:**
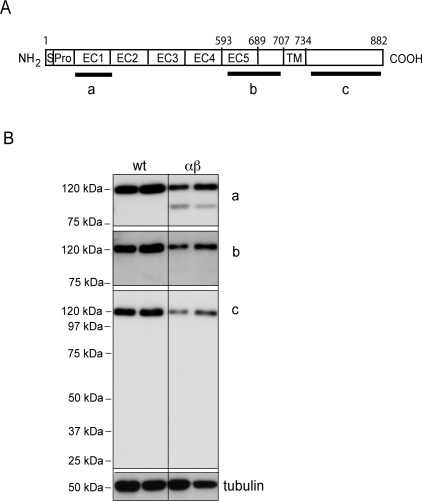
Molecular mapping of the cleavage site of E-cadherin by meprinβ. (A) Schematic representation of E-cadherin and antibody epitopes. *a*: DECMA, *b*: SC7870, *c*: BDclone 36. S: Signal peptide, Pro: Propeptide, EC: Cadherin-domain, TM: transmembrane domain. (B) Protein extracts from MDCKwt and MDCKαβ cell lines were resolved by SDS-PAGE and analyzed by immunoblot using antibodies *a–c*. The same membrane was successively stripped and reprobed.

To define the cleavage site of E-cadherin by meprinβ, immunoblots of MDCKwt and MDCKαβ lysates were reprobed on the same blot with three different region-specific antibodies (Figure 4A). The mature form of E-cadherin and the 97-kDa fragment were both detected by the N-terminal DECMA antibody (Figure 4B). The SC7870 antibody, raised against the last extracellular domain of E-cadherin (EC5) and the juxtamembranous region, did not detect the 97-kDa fragment under conditions that revealed an equally strong signal for the mature 120-kDa form of E-cadherin as seen with the DECMA antibody. This indicated that SC7870 epitopes are lost in the 97-kDa fragment. Considering the size of the cleaved fragment, it can be concluded that the cleavage site is located close to the epitopes covered by the SC7870 antibody and in vicinity of the transmembrane domain. Consistently, the cytoplasmic BD36 antibody failed to detect the 97-kDa E-cadherin fragment, or any smaller residual fragment (Figure 4B), further indicating that the cytosolic tail of E-cadherin may be degraded after cleavage by meprinβ.

### The 97-kDa E-cadherin fragment is not released from cells

**Figure 5:**
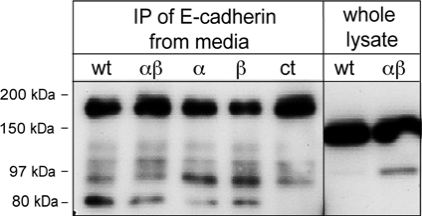
The 97-kDa E-cadherin fragment is not released from cells. E-cadherin was immunoprecipitated with the N-terminal antibody (DECMA) from conditioned media of MDCK cells. Fresh medium was used as a negative control (ct). Whole cell lysates were loaded on the same gel as reference. A soluble 80-kDa E-cadherin fragment was detected in conditioned media irrespective of meprin expression (arrowhead). No specific signal was detectable at 97-kDa. Additional bands were present in all immunoprecipitations including the fresh medium sample and thus were attributable to background.

Cleavage of E-cadherin by meprinβ may lead to the release of a soluble fragment in culture media. We therefore immunoprecipitated E-cadherin from conditioned culture media of confluent cell cultures (Figure 5). MDCK cells constitutively released small amounts of a 80-kDa E-cadherin fragment irrespective of the presence of meprin. However, the 97-kDa E-cadherin fragment was not detectable, indicating that it may be retained by the cells.

### E-cadherin processing by meprinβ affects its cytoplasmic binding partners β-catenin and plakoglobin but not α-catenin

**Figure 6:**
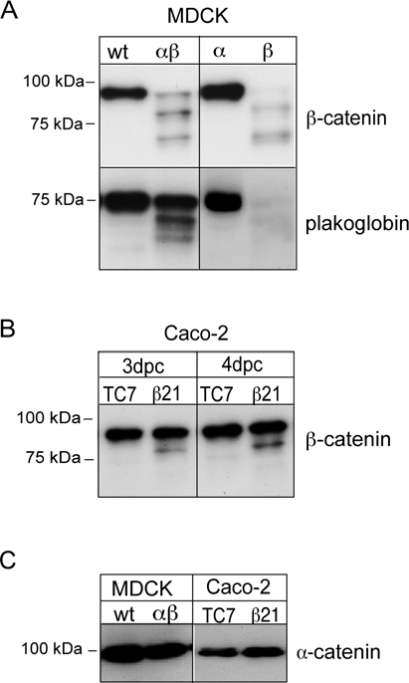
The direct binding partners of E-cadherin are processed in MDCK and Caco-2 cells expressing meprinβ. β-catenin and plakoglobin were analyzed in protein extracts of MDCKwt, MDCKαβ, MDCKα and MDCKβ cells (A), as well as in Caco-2-TC7 and Caco-2-β21 cells (B). Two cleaved fragments were detected in lysates of cells expressing meprinβ. α-catenin remained intact (C).

The assembly of E-cadherin with the catenin proteins precedes the linkage of the adherens junctions to the cytoskeleton, which participates in the stabilization of cell-cell adhesions [38]–[40]. We investigated whether E-cadherin cleavage affected their integrity. Figure 6 shows that in MDCK and Caco-2 cells expressing meprinβ, β-catenin and plakoglobin were both cleaved into two smaller fragments (Figure 6A and B). In contrast, α-catenin was not affected in meprinβ-expressing cells (Figure 6C).

### Uniform distribution of meprinβ over the cell surface in non-polarized MDCK cells

**Figure 7:**
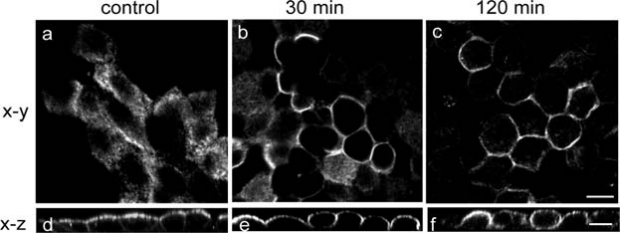
Meprinβ distribution in polarized and depolarized MDCKαβ cells. MDCKαβ cells were grown to confluence on filter supports in complete medium and subjected to a Ca^2+^-switch (described in experimental procedures). After fixation and immunostaining, meprinβ localization was analyzed by CLSM. Pictures (*a–c*) are single optical sections (x–y). Corresponding orthogonal sections in the x–z plane are shown below (*d–f*). Picture *a* shows meprinβ localization in confluent polarized MDCKαβ cells. Pictures *b*+*e* and *c*+*f* show meprinβ relocalization 30 minutes and 120 minutes after removal of extracellular Ca^2+^. Bar = 10 µm.

To further confirm the possibility of meprinβ localization to lateral membranes, we determined meprinβ distribution upon perturbation of cell-cell adhesion and cell polarization after withdrawing Ca^2+^ from the medium. MDCKαβ cells were grown to confluence, immunostained with a meprinβ-specific antibody, and analyzed by confocal microscopy. Meprin localization in cells grown in Ca^2+^-containing medium (control condition) was compared with cells exposed to Ca^2+^-free medium for 30 minutes and 120 minutes. Initially, meprinβ appeared at the apical membrane domain only (Figure 7, panels a and d), which is in accordance with previous biochemical studies demonstrating an apical targeting of this isoform [5]. After 30 minutes of incubation in Ca^2+^-free medium, the cells rounded up and meprinβ relocalized to lateral membrane domains (Figure 7, panels b and e). After 120 minutes, meprinβ redistributed all over the plasma membrane (Figure 7, panels c and f). After 6 hours of recovery in normal medium, meprinβ relocalized to the apical membrane domain (not shown). These results exemplify that meprinβ may distribute all over the cell surface upon loss of cell polarization, which may expose additional substrates that normally would be inaccessible to the protease. Such potential substrates include proteins that establish cell-cell contacts. Therefore, in the presence of meprinβ cleaving E-cadherin (see above), the formation of cell-cell contacts may be affected.

### Reduced cell-cell adhesion and formation of multicellular aggregates in meprinβ-expressing cells

**Figure 8:**
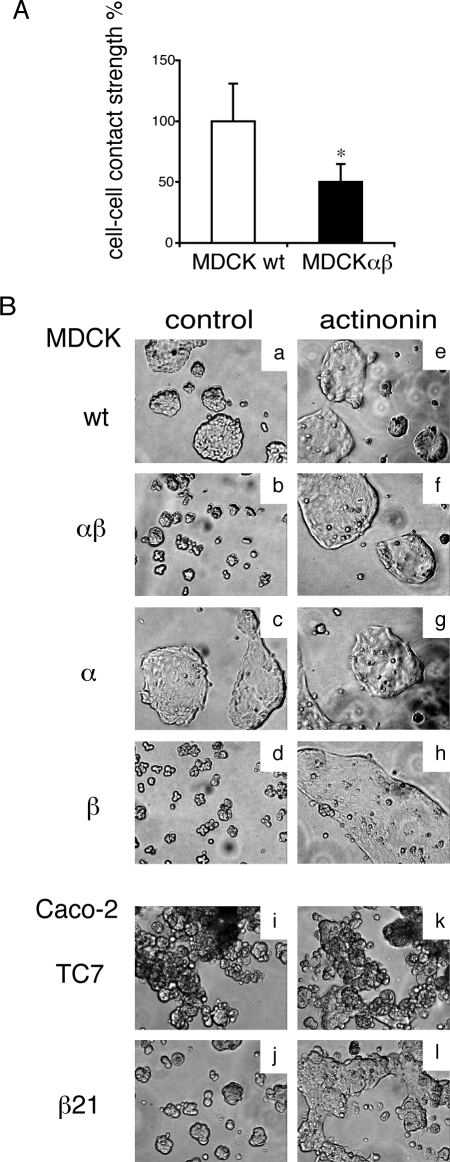
Meprinβ affects intercellular adhesion. (A) Cell-cell contact strength was measured using the dispase assay (described in experimental procedures). The graph shows the mean values +/− SD from 3 independent experiments. * = p<0.05. (B) Cell aggregation assay with MDCK cell lines (*a–h*) and Caco-2 cell lines (*i–l*). Hanging drops of cell suspensions were incubated overnight. Representative pictures of three independent experiments with each condition in 6 replicates are shown. Cells expressing meprinβ form smaller aggregates (*b*, *d*, *j* compared to *a*, *c*, *i*). The presence of actinonin (right panels) in the resuspension medium reverted the phenotype of meprinβ expressing cells (*f*, *h*, *l*) without having an effect in the other cell lines (*e*, *g*, *k*).

To determine the functional consequence of meprinβ-mediated E-cadherin for intercellular adhesiveness, we performed two different assays. To measure the adhesive strength between cells, we applied mechanical stress to a cell monolayer detached with dispase [34]. Figure 8A shows that the adhesive strength between MDCKαβ cells was significantly decreased by 50% as compared to MDCKwt cells. We next evaluated the effect of meprinβ on aggregation of suspended cells in a hanging drop assay [35]. MDCKwt and MDCKα cells formed large aggregates, whereas much smaller aggregates were observed with MDCKαβ and MDCKβ cells (Figure 8B, compare panels *a* and *c* to *b* and *d*). To confirm the implication of meprinβ in the reduction of cell-cell aggregation, we repeated the experiment in the presence of the meprin inhibitor actinonin. Under these conditions, MDCKαβ and MDCKβ cells formed aggregates with a size comparable to MDCKwt and MDCKα cells (Figure 8B, panels *e–h*). Identical results were observed with Caco-2 wild-type and meprinβ–expressing cells (see Figure 8B, panels *i*–*l*). These findings demonstrate that intercellular adhesion is reduced in meprin β-expressing epithelial cells.

## Discussion

Previous studies indicate an involvement of the metalloprotease meprin in inflammation and cancer [41]. However, the molecular mechanisms implicating meprin in these processes are unknown. Here, we provide evidence that meprinβ targets and processes E-cadherin thus affecting intercellular adhesion. In particular, our study shows that, in two different epithelial cell types, the expression of meprinβ induced the cleavage of E-cadherin, generating a specific 97-kDa fragment. Cleavage of E-cadherin was enhanced upon *in situ* activation of cell-surface expressed meprin by limited trypsin treatment, and inversely decreased after inhibition of meprin by actinonin, indicating that the proteolytic activity was required for cleavage of E-cadherin.

We demonstrate the direct and specific cleavage of E-cadherin in an *in vitro* cleavage assay with purified recombinant active meprinβ. When compared with two other proteases, MMP-7 and ADAM-10, which are both expressed in epithelial cells and previously have been shown to target E-cadherin [29], [30], only meprinβ yielded the 97-kDa fragment, which therefore appears to be a specific cleavage product. ADAM-10 and MMP-7 both produced slightly larger fragments. In a very similar *in vitro* cleavage assay reported previously, MMP-7 generated several cleavage products of E-cadherin with apparent molecular weights down to 80 kDa [29]. However, the enzyme concentrations used in that reported experiment (minimum concentration 20 ng/100 µl) were significantly higher than in our assay (maximum concentration 1.25 nM equals 3.5 ng/100 µl). With the low enzyme concentrations used in this study, MMP-7 generated only a single product. Therefore, MMP-7 displays variable cleavage efficiency at different sites in E-cadherin and we may have missed less efficient cleavage sites that would yield the additional MMP-7 specific fragments. Thus, multiple proteases may target a range of sites in E-cadherin with variable cleavage efficiencies, which co-determine the final E-cadherin cleavage products.

In contrast to meprinβ, meprinα was not implicated in the cleavage of E-cadherin, as the 97-kDa E-cadherin fragment was not detected in lysates of cells expressing only the α subunit. This was also supported by the failure of purified recombinant active meprinα to generate the 97-kDa E-cadherin fragment *in vitro* (not shown). Different substrate and peptide bond specificities between the two isoforms have been previously described [13], and their differential activity towards E-cadherin corroborates the distinct functions of both subunits.

The cleavage of E-cadherin by meprinβ is unique. Although truncated E-cadherin fragments with similar size were described in several reports, these were generated by intracellular proteases [42]–[44]. In contrast, we defined the cleavage site in the ectodomain close to the plasma membrane (see Figure 4). This is consistent with our observation that a recombinant soluble form of E-cadherin lacking the transmembrane region was not cleaved by meprinβ *in vitro* (not shown). It is also consistent with a structural model of meprinβ that has been proposed recently on the basis of cross-linking experiments [45]. According to this model, meprinβ folds back onto itself, positioning the catalytic domain close to the plasma membrane [45].

Prior studies have shown the extracellular cleavage of E-cadherin by different metalloproteases, which led to the release of soluble fragments. A soluble 95-kDa E-cadherin fragment generated by an unidentified metalloprotease was found in cultured cancer cells [44]. In prostate and breast cancer cells, matrilysin (MMP-7) and stromelysin (MMP-3) have been shown to generate a soluble 80-kDa fragment [29], [37], and ADAM-10, a transmembrane protease, controls constitutive and regulated shedding of E-cadherin in cell cultures of keratinocytes and *in vivo* in mouse embryos [30]. Small amounts of the 80-kDa E-cadherin are also shed from MDCK cells, which was not affected by meprin (Figure 5) and possibly is mediated by endogenous ADAM-10 targeted to adherens junctions [46]. In contrast, our data indicate that the 97-kDa E-cadherin fragment is retained by the cells, where it possibly interferes with the function of full length E-cadherin at the cell surface. For example, the 97-kDa fragment might modulate the interaction between E-cadherin subunits, thereby affecting mechanical links as well as intracellular signalling pathways. There is increasing evidence that E-cadherin triggers intracellular pathways implicated in growth arrest and differentiation, which depend on the formation of homophilic bonds at the cell surface [47]–[49]. In addition, cleavage and subsequent degradation of the E-cadherin cytoplasmic tail may directly prevent activation of signaling cascades that depend on the recruitment of cytoplasmic effectors, such as phospatidylinositol-3 kinase, to the E-cadherin tail.

The processing of E-cadherin by MMPs or ADAM-10 has been shown to lead to the generation of cell-associated C-terminal E-cadherin fragments of approximately 40 kDa, which was accompanied by increased β-catenin-mediated signaling. In contrast, no such fragment was detectable with a C-terminal antibody in meprinβ-expressing MDCK cells, indicating that it is prone to degradation. We also show that in parallel with the disappearance of the C-cytosolic tail of E-cadherin in meprinβ-expressing cells, the intracellular binding partners β-catenin and plakoglobin were also processed, but not α-catenin, which is not directly linked to E-cadherin. Previous reports have shown that these proteins are cleaved by caspases 3 and 8 [50] during apoptosis, and by calpain in prostate and mammary tumor cells [51], and it is therefore possible that these intracellular proteases are implicated in meprinβ-expressing MDCK cells as well.

As a consequence of the disruption of the cadherin/catenin complex, the intercellular adhesion in meprinβ-expressing cells was significantly weakened, as demonstrated by the decrease in cell-cell contact strength and cell-cell aggregation. The 97-kDa E-cadherin fragment itself, the degradation of the cytoplasmic region of E-cadherin after cleavage by meprinβ, as well as the specific proteolytic processing of its direct binding partners may account for the impaired cell-cell adhesion. This is supported by several reports where the absence of the catenin binding domain was shown to abolish the adhesive activity of E-cadherin [39], [52].

The transition from preconfluent to confluent cell cultures recapitulates the development of epithelial cell polarity and concomitant sequestering of proteins to specific membrane domains [53]. This explains the different localization patterns of meprinβ and E-cadherin during progression to fully established cell polarity and has direct implications for the cellular compartment of meprinβ-mediated E-cadherin processing. In subconfluent cultures of incompletely polarized MDCK cells both partners colocalize at lateral membranes, where E-cadherin processing may occur (Figure 3). However, in confluent cultures of fully polarized cells, the 97-kDa fragment is also present, despite the spatial restriction of meprinβ and E-cadherin to separate membrane domains. As the formation of a stable protein complex between a protease and its substrate is an exception, we speculate that processing of E-cadherin in polarized cells occurs in a transient protein complex in the absence of detectable colocalization at the microscopic level. Sorting of E-cadherin in polarized cells displays a high degree of plasticity. For instance, direct delivery of E-cadherin to lateral membrane in MDCK cells occurs only two days after reaching the confluent stage, and before that, a substantial part of the newly synthesized E-cadherin pool is delivered to and transiently resides at the apical side [53]. Therefore, the cellular sorting routes for E-cadherin and meprinβ overlap substantially in polarized MDCK cells, which may facilitate their transient interaction.

This is the first report implicating meprinβ in the control of the adhesive function in epithelial cells. The regulation of epithelial cell-cell adhesion is of utmost importance to maintain tissue architecture and to prevent cell invasion during pathological conditions [19], [54], and E-cadherin is a primordial factor often repressed during transition from adenoma to carcinoma [20], [55]. Therefore, cleavage of E-cadherin by meprinβ may be a means to reduce E-cadherin-mediated cell-cell-interactions. The redistribution of meprinβ to the basolateral membrane of MDCK cells upon Ca^2+^-switch-induced depolarization is of interest, because the polarization status of epithelial cells is perturbed in diverse pathological conditions, including ischaemic disease, inflammation and cancer. An aberrant targeting of meprinβ in such conditions may have important functional consequences as it may enhance access to basolateral substrates, including E-cadherin. A cancer-specific meprinβ transcript, meprinβ′, has been shown to be expressed from an alternative promoter region that includes multiple oncogene binding sites in cancer cells of mammary, colonic and pancreatic origin [18], [56]. Hence, the expression of meprinβ in cancer coupled to perturbed protein sorting and high E-cadherin processing efficiency indicate that this protease can modulate cancer cell functions.
